# Mycosis of the Trachea

**Published:** 1881-08

**Authors:** 


### Mycosis of the Trachea.

A German physician, Dr. Hertenrich, has lately cured a case of this rare affection by iodine inhalations used thrice daily for two weeks. Carbolic inhalations totally failed to do good. Parasitic affections of the ear and nose are of more frequent occurrence than of the air-passages. In all these cases the questions arise: Is the parasite a cause or a consequence of disease? or is it first a consequence and next an aggravation? or is it of itself the sole source of the morbid phenomena?

